# COVID-19: The First 30 Days at a UK Level 1 Trauma Centre and Lessons Learnt

**DOI:** 10.7759/cureus.11547

**Published:** 2020-11-18

**Authors:** Paul A Andrzejowski, Anthony Howard, James Shen Hwa Vun, Nauman Manzoor, Nikolaos Patsiogiannis, Nikolaos K Kanakaris, Peter V Giannoudis

**Affiliations:** 1 Trauma and Orthopaedics, Leeds Teaching Hospitals NHS Trust, Leeds, GBR

**Keywords:** covid-19, orthopedics and trauma, hospital epidemiology, biomarker, lessons learnt

## Abstract

Aims

To analyse the learning points from the first 30 days of the COVID-19 lockdown at our institution.

Patients & methods

Following ethical approval, data were collected prospectively on all patients admitted under orthopaedics between March 23, 2020, and April 22, 2020. This included baseline demographics (sex, age), biochemical (blood tests), radiological (chest X-ray (CXR), computed tomography (CT)), nature and mechanism of injury, comorbidities, regular medication, observations, specific respiratory symptoms of COVID-19, management, operations, time to theatre, and outcome including mortality incidence. The nature of injury and operations performed were compared to the same period of the previous year (2019).

Results

During the study period, 162 (74 males) patients were admitted, with a mean age of 60.7 (range 1-101, SD 2.1). On admission, 66 (41%) patients were tested for COVID, out of which eight (13.7%) patients tested positive. Subsequently, another four patients tested positive, who developed symptoms after admission. Four out 12 (33%) confirmed COVID patients died. During this period, 4/150 other patients also died of other causes (mortality incidence 2.6%). The average ages of COVID non-survivors vs survivors were 88, SD 1, vs 76, SD 12, respectively; 2/4 had concurrent diabetes and cancer, another cancer alone, and another complex autoimmune disease managed by immunosuppressive medication.

Overall admissions significantly reduced by almost 50% compared with the previous year (162 vs 373, p=<0.05), including cases of polytrauma (15 vs 33). Time to surgery was increased by an average of one day, mainly due to time taken for COVID-19 swab results to come back, and in positive patients, this was an average of 2.75 days (0-13). Lymphopenia was a useful biomarker of COVID, with levels significantly different between groups (p=<0.05). Of the clinical symptoms assessed, 8/12 patients experienced positive chest symptoms or pyrexia but only four had positive CXR changes.

Discussion & lessons learnt

Eight out of 12 patients who contracted COVID-19 survived without needing intensive care. Non-survivors were older with significant comorbidities. Lymphopenia is a good biomarker of the disease, but suspicious CXR was not sensitive for excluding it. Trauma volume reduced. We have highlighted significant changes to expect should there be a second wave of the virus. Key lessons learnt were that reduction in trauma volume and cessation of elective operating allowed for redeployment, including taking over the minor injury unit; more senior, consultant decision-makers ‘at the front door’ reduced unnecessary admissions. Increased use of conservative practice was effective at reducing operations required. Expedited COVID swab test processing allowed early de-escalation of isolation, reducing time to surgery. We expect approximately 12% of the typical orthopaedic population to be admitted with COVID, and up to 33% of these patients to die within 28 days of contracting the virus. The vast majority of patients, however, can be managed appropriately with ward-level care. An early decision on escalation and resuscitation status in the emergency department improves patient flow significantly. Remote working was effective and could be extended in the future.

We have highlighted the significant changes to expect should there be a second wave of the virus and effective solutions for managing the problems that arise, which could be useful for other units.

## Introduction

From January 2020, the demands and delivery of health care internationally changed significantly due to the COVID-19 virus. Late in 2019, 27 patients suffering from pneumonia of unknown aetiology were being treated in Wuhan City, China. The patients presented with a dry cough, dyspnoea, fever and bilateral lung infiltrates on chest radiograph [1]. The common denominator in these patients was a seafood wholesale market, trading in fish and a number of live animal species, including bats, marmots and snakes [2]. By January 7, 2020, the Chinese Centre for Disease Control and Prevention had identified the causative agent, the World Health Organisation (WHO) had named the disease COVID-19 [3]. COVID-19 is a zoonotic virus that causes severe acute respiratory syndrome [4].

Within a short period of time, the disease spread from China at a global scale, prompting the WHO to declare a state of pandemic. In the UK, the first case of the disease was diagnosed on January 28, 2020, in two patients that had arrived from China to stay in a hotel in York [5-6]. The UK Government introduced a series of measures to attempt to reduce the rate of transmission and on March 23, 2020, which implemented strict measures by ordering individuals to stay at home unless they fell within a restricted number of circumstances, which became known as the “Lockdown” [7].

The purpose of the study was to comparatively assess the nature of the change in admissions within a level 1 major trauma centre in the UK during the first 30 days of the UK National lockdown. This prospective study was undertaken to guide the hospital’s initial response. Then, through a detailed analysis of the catalogued experience, to derive lessons that could be applied if there were to be a second spike of infections.

## Materials and methods

A prospective cohort study of patients admitted to our institution from the start of the ‘lockdown’ on March 23 for the first 30 days until April 22, 2020 (inclusive) was undertaken. All patients admitted due to an orthopaedic injury or trauma-related reasons were included: paediatric, general and major trauma patients.

The data collected included: demographics (sex, age), biochemical (blood tests), and radiological (chest X-rays (CXR) and computed tomography (CT)) investigations, as well as clinical information (nature and mechanism of injuries, comorbidities, regular medication, observations, the potential symptoms of COVID on or during admission, investigations for COVID-19), management (whether higher-level care was required, time to theatre and operations performed) and outcomes (mortality, complications).

The comorbidity data were categorised in accordance with the NHS guidance on pre-existing conditions that increased the mortality risk to individuals [8]. These were then subcategorised into respiratory illness (chronic obstructive pulmonary disease (COPD), asthma, idiopathic pulmonary fibrosis, other lung fibrosis, cystic fibrosis (CF), and other lung pathology), cardiovascular morbidity (hypertension, ischaemic heart disease, heart failure, atrial fibrillation), neurological comorbidity (stroke, multiple sclerosis, Parkinson’s disease, other neuromuscular pathology), haematological pathology (haematological malignancy, other haematological pathology, bone marrow or stem cell transplant in the last 6 m), auto-immune conditions (rheumatoid arthritis, other auto-immune diseases, diabetes), organ pathology (kidney disease, organ transplant, liver disease, spleen pathology, previous splenectomy, thyroid disease), factors that may alter immunity (human immunodeficiency virus (HIV), low vitamin D), and pregnancy, as well as other forms of cancer. Other comorbidities were also recorded where they occurred.

The nature of the injuries and types of treatments undertaken during the first 30-days of the COVID-19 pandemic was compared to the previous equivalent timescale in 2019. Polytrauma was classified as at least two injuries (AIS -3) in two different body regions [9]. Where there was more than one injury, the most dominant injury was selected for classification purposes.

Following this, data were synthesised, and IBM SPSS Statistics for Windows, version 26 (IBM Corp, Armonk, New York) was used to perform statistical analysis on the relevant sections of data. Descriptive statistics were calculated and presented, Kruskal-Wallis analysis of variance (ANOVA) was used to assess non-parametric data, and the student's T-test for normally distributed data; findings were deemed to be statistically significant where p ≤0.05.

## Results

During the first 30 days, 162 patients were admitted (74 male/88 female), mean age 60.7 (range 1-101, SD 29.1), as shown in Table 1. Overall, on admission, 66 (41%) patients were tested for COVID, out of which eight (13.7%) patients tested positive. Subsequently, one patient who tested negative on admission tested positive and out of the group of patients that were not tested (96), three patients tested positive.

**Table 1:** COVID status of patients admitted during the first 30 days o/a: on admission; CXR: chest X-ray

COVID-19 status group	Age: Mean, range. (years)	Sex M:F (n)	Admission COVID-19 status (n)	Subsequent COVID-19 tests (n)	Subsequent COVID-19 +ve status change (n)	Cough o/a (n)	Temp >37.5 o/a (n)	COVID-19 CXR changes o/a (n)	Subsequent COVID-19 CXR changes (n)
Confirmed Positive	79 (58-90, SD 11.9)	6:5	8	NA	NA	3	4	3	0
Confirmed Negative	68 (2-98, SD 23.9)	30:43	58	8	1	1	8	3	1
Untested (Low suspicion)	46 (1-98, SD 30.9)	37:42	96	19	3	8	0	0	4 (2/4 COVID +ve)

Initially, in our institution, when the pandemic began, we were using an in-house RdRp SARS-CoV-2 polymerase chain reaction (PCR) assay rolled out nationally by Public Health England. We switched over to the commercial RealStar® SARS-CoV-2 RT-PCR Kit (from Altona Diagnostics, Hamburg, Germany) on April 6, 2020, and then over to the Hologic Panther TMA assay (a commercial end-to-end random-access platform; Hologic, Inc., Marlborough, Massachusetts) on May 14, 2020. We do, however, still use the RealStar® SARS-CoV-2 RT-PCR Kit for lower respiratory tract samples.

COVID status on admission

Initial swabs positive group: Eight patients swabbed positive on admission. Four had cough or pyrexia, and five were lymphopenic. Three out of the eight had CXR changes. Two (25%) of these patients died; radiologically, they had COVID-consistent CXR changes (one of these patients required ventilation on intensive treatment unit (ITU)) (Tables 1-2). Compared with non-COVID patients, two others also required ITU admission; both following head injury.

**Table 2:** Summary of COVID-positive patients (patients 1-8 positive o/a, patient 9 negative o/a, patients 10-12 not tested o/a) *See reference to this patient’s mortality status in text. ¥ Died following discharge from orthopaedics PCR: polymerase chain reaction; CXR: chest X-ray; ITU: intensive care unit; CPAP: continuous positive airway pressure therapy; COPD: chronic obstructive pulmonary disease; DM: diabetes mellitus; SOB: shortness of breath; TKR: total knee replacement; HTN: hypertension; CT: computed tomography; CVA: cerebrovascular accident; DAIR: debridement, antibiotics, irrigation, and retention; WCC: white cell count; DHS: dynamic hip screw; o/a: on admission; IT: intertrochanteric; IC: intracapsular, GT: greater trochanter; sx: symptoms.

ID	Age	Sex	Out-come	MOI/Pathology	Injury	Comorbidity	PCR swab reason or positive symptoms	Lymph	CXR COVID change o/a?	Subsequent CXR change	ITU?	CPAP?	Ventilated?	Operation	Days to theatre
1 (+veo/a)	88	F	Died ¥	Mechanical fall	Open ankle #	COPD, DM, Lung Ca	Cough, SOB, wheeze, pyrexia	1.28	YES	YES	No	No	No	Fixation	2 (a/w PCR)
2 (+veo/a)	60	M	Died	Cellulitis	Calf abscess	Mixed auto-immune disease	Exposed to known COVID, pyrexia, cough	1.57	YES	NA	YES: COVID	YES: COVID	YES: COVID	Debridement	2 (a/w PCR)
3 (+veo/a)	82	F	Survived	Mechanical fall	GT #	Stoke, epilepsy	Lymphopenia, never pyrexia or chest sx	0.50	No	NA	No	No	No	Nil	NA
4 (+veo/a)	90	F	Survived	Mechanical fall	IC hip #	Hypertension	Exposed to known COVID	1.41	No	NA	No	No	No	Hemiarthroplasty	2 (a/w PCR)
5 (+veo/a)	58	F	Survived	Hot swollen knee	Infected TKR	HTN, hypothyroid	Raised WCC and CRP, precaution: swab pre-theatre, asymptomatic	0.69	No	NA	No	No	No	DAIR	0
6 (+veo/a)	64	M	Survived	Mechanical fall	Tibial plateau #	Organ transplant	Pyrexia, abnormal CXR, mixed COVID/bacterial pneumonia	0.55	YES	NA	No	No	No	Ilizarov frame	13 (a/w PCR, unwell)
7 (+veo/a)	81	M	Survived	Mechanical fall	IC hip #	Bladder cancer	Raised WCC, cough, pyrexia	0.64	No	No	No	No	No	Hemiarthroplasty	3 (a/w PCR)
8 (+veo/a)	84	M	Survived	Mechanical fall	IT hip #	Hypertension, CVA, lung disease	o/a: Cough, Pyrexia	0.73	No	NA	No	No	No	DHS	2
9 (-ve o/a)	84	M	Survived	Mechanical fall	Pelvic, clavicle #	Atrial fibrillation	Abnormal CXR and CT a +ve PCR 4/7 after admission	0.40	No	YES	No	No	No	Nil	NA
10 (no test o/a)	88	F	Survived *	Mechanical fall	Pubic ramus #	COPD, atrial fibrillation, low Vit D	Hypoxia, SOB, pyrexia following admission a+ve PCR 6/7 after admission	0.45	NA	YES	No	No	No	Nil	NA
11 (no test o/a)	87	M	Died ¥	Mechanical fall	IC hip #	Prostate Ca mets, OA	Abnormal CXR, lymphopenia. Developed pyrexia and chest sx days after swab, a +ve PCR 13/7 after discharge, readmitted under a different specialty	0.74	YES	NA	No	No	No	IMN	1
12 (no test o/a)	89	F	Died ¥	Mechanical fall	IT hip #	Diabetes, lymphoma	Diarrhoea/Vomiting à+ve 5/7 after discharge	0.65	No	No	No	No	No	DHS	0

Initial swabs negative group: One patient tested negative on admission but went on to develop symptoms with a normal CXR, tested positive four days later, and survived to discharge (Tables 1-2).

No swab on admission group: Out of the 96 non-tested patients, three went on to have positive COVID tests subsequently. Moreover, two out of three developed a CXR consistent with COVID (Tables 1-2). Out of these three patients, two died (one whilst an inpatient; the other one developed symptoms five days after discharge back to their nursing home, dying four days later). Fourteen other patients were also subsequently tested and found to be negative. None of these patients had concerning chest X-rays on admission.

Mortality in COVID patients: Four patients who tested positive for COVID died. The mean time to death was 8 days (4, 6, 10, 12 days, respectively, 2:2 M:F, mean age 88: range 87-89, SD 1). Mortality incidence was 4/12 (33%), as shown in Table 2.

Mortality in non-COVID patients: Four patients died (2:2 M:F, mean age 83.5: range 75-91, SD 7). The mean time to death was 25.25 days (13, 25, 29, 34 days, respectively). The causes of death included septicaemia from parotid gland infection, non-COVID pneumonia, and traumatic intracranial haemorrhage (this was the only patient to die from their injuries). Mortality incidence was 4/150 (2.6%), as shown in Table 3.

**Table 3:** Deaths in non-COVID patients COPD: chronic obstructive pulmonary disease; DHS: dynamic hip screw; HTN: hypertension; CVA: cerebrovascular accident; T2DM: type 2 diabetes mellitus; CKD: chronic kidney disease; THR: total hip replacement; MUA: manipulation under anaesthesia; o/a: on admission

ID	Age	Sex	Cause of Death	MOI	Injury	Comorbidity:	Operation	Days to Theatre
13	75	M	Non-COVID pneumonia (COVID -ve o/a)	Mechanical fall	IT hip #, Closed wrist #, Traumatic SAH	COPD, lung cancer, stroke	DHS	16 (unwell)
14	81	M	Traumatic injuries (COVID -ve o/a)	Fell down a flight of stairs	Shoulder #, C-spine #, Other spine #, Extensive intracranial haemorrhage.	Right-sided CVA, HTN, T2DM, hypothyroidism, IHD	Nil	NA
15	87	F	Parotid infection: septicaemia (COVID -ve following admission)	Mechanical fall	Dislocated THR	Heart failure, atrial fibrillation, diabetes, CKD	MUA hip	1
16	91	F	Died 13 days after discharge, records unavailable (COVID -ve o/a)	Mechanical fall	IC hip #	Ischaemic heart disease	Hemiarthroplasty	2

Number and type of admissions COVID and pre-COVID time periods

**Figure 1:**
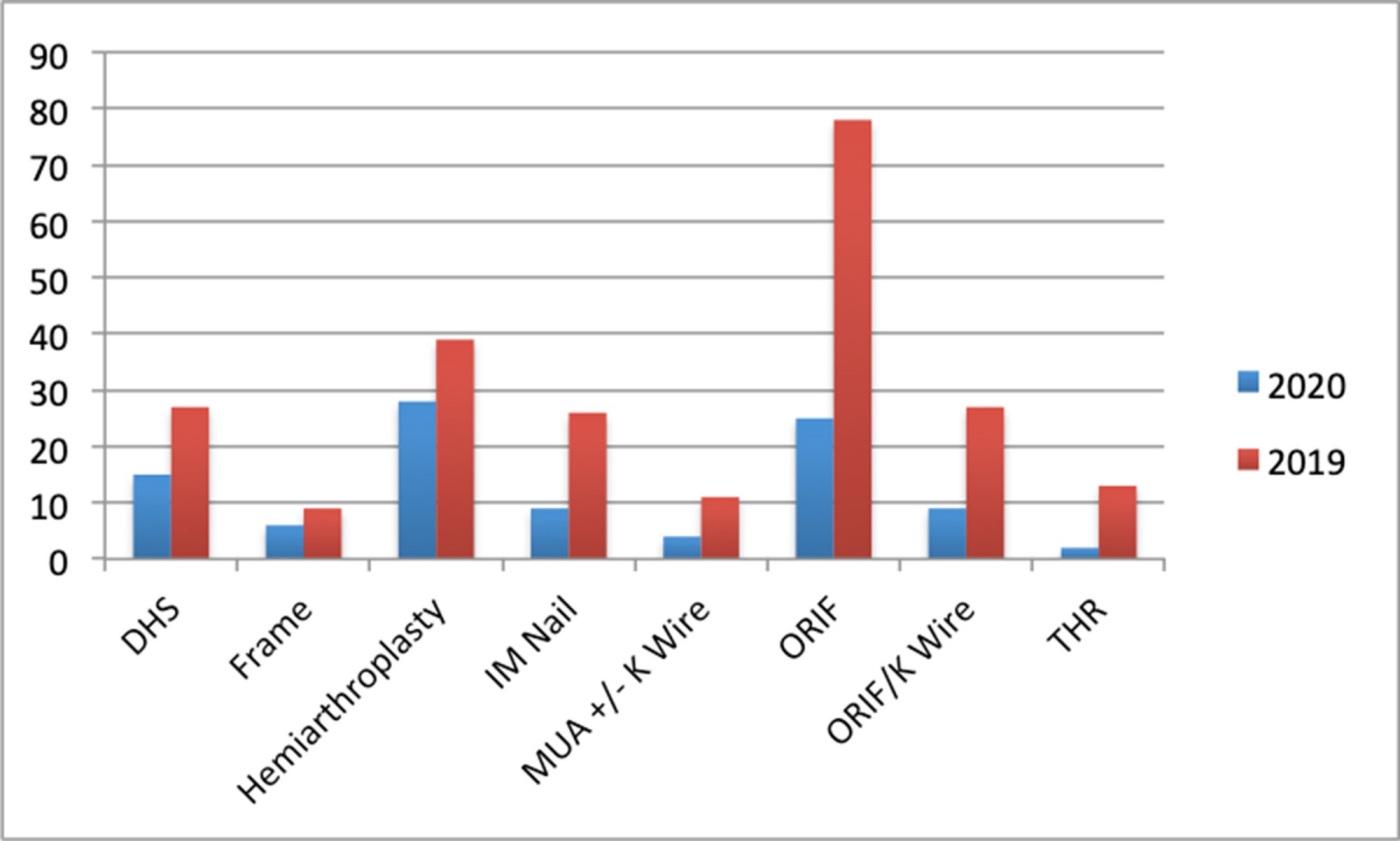
Comparison of the number of surgical procedure types between the first 30-day period and the same period the previous year

When the first 30-day period was compared to the same time period of the previous year (2019), it was noted that the number of admissions was reduced by almost 50% (COVID period 162 versus 373 pre-COVID period), as shown in Table 4. The nature of the injuries was significantly different (p<0.05), except for septic arthritis. Furthermore, there were significant differences in the number of certain surgical interventions undertaken, as shown in Figure 1.

**Table 4:** Comparison of type injury between the first 30-day period and the same period the previous year

Type of injury		First 30 days of COVID 2020	March 23 to April 22, 2019	Difference
Polytrauma		15	33	- 18
Dislocation		5	9	- 4
	Naïve Joint	2	3	- 1
	Arthroplasty	3	6	- 3
Fractures		108	248	- 140
	Hip	56	82	- 26
	Periprosthetic	2	10	- 8
	Thigh/Knee/Tibia	18	45	- 27
	Ankle	16	32	- 16
	Foot	2	4	- 2
	Forearm/Wrist	3	36	- 33
	Elbow/Humerus	6	20	- 14
	Pelvis	5	19	- 14
Soft tissue		9	30	- 21
Infection		11	15	- 4
	Septic Arthritis	6	8	- 2
	Arthroplasty Infection	2	2	0
	General Soft/Osteomyelitis	3	5	- 2
SUFE		1	4	- 3
Other		13	34	- 21

Biochemical/haematological profile and clinical symptoms of COVID-positive patients

The biochemical/haematological markers tested on COVID-positive and COVID-negative patients are shown in Table 5. The level of lymphocytes was significantly different (p=0.01) in patients with a positive COVID-19 swab. However, the ferritin level in the positive patients was not significantly higher as compared to the COVID-negative group (p>0.05), as shown in Table 5. There was no significant relationship between COVID status and the 32 co-morbidities recorded amongst all the patients admitted (p>0.05).

**Table 5:** Comparison of different blood markers in COVID-positive and negative patients * p = <0.05, † p = 0.55 Hb: haemoglobin; MCV: mean corpuscular volume; WBC: white blood cells; CRP: C-reactive protein

	COVID Positive Test (12 patients)	COVID Negative Test (73 patients)	Untested (77 patients)
Hb	110 (87-130, SD 14.3)	123 (83-215, SD 25.7)	128 (70-162, SD 16.8)
MCV	91 (74-100, SD 8.2)	93 (75-111, SD 6.4)	91 (75-105, SD 6.3)
WBC	11.5 (4.1-28, SD 7.1)	12.2 (3-23, SD 4.2)	9.6 (1.2-18, SD 3.4)
Neutrophils	11 (3-17, SD 4.8)	9.8 (1-20, SD 3.9)	7.4 (0.89-14, SD 3.2)
Lymphocytes	0.8 (0.40-1.6, SD 0.4) *	1.29 (0.32-4.5, SD 0.80) *	1.45 (0.40-4, SD 0.75)
Platelets	222 (116-453, SD 108.5)	269 (42-758, SD 129)	269 (132-518, SD 87)
CRP	128 (11-473, SD 164.3) †	88 (1-469, SD 109) †	
Ferritin	584 (23-1661, SD 932)	452 (12-2828, SD 699)	
Ca	2.2 (2.1-2.4, SD 0.07)	2.3 (2-2.69, SD 0.14)	
Vitamin D	51 (25-102, SD 43.9)	58 (20-171, SD 33.4)	

When looking at the relationship of symptoms to positive swab results, most patients (8/12) exhibited positive chest symptoms or pyrexia (Table 2).

Time to surgical intervention

Time to theatre was an average of two (range 0-13) days in patients who underwent a COVID-19 PCR swab and one day (range 0-4) in patients who were not tested. In those who tested positive, however, time to theatre was even longer (2.75 days, range 0-13), as their operations could only be performed in a specially equipped ‘hot’ theatre.

Place of residence in older patients (care facility vs own home)

In Table 6, we can see the proportions of patients who were admitted from care facilities (nursing homes and residential homes vs. their own home). We have broken this down by COVID and non-COVID patients; in our relatively small cohort, there is little difference overall between a positive COVID swab diagnosis on admission to hospital and a care facility residence. When examining the social situation of patients with hip fractures in itself, which form the majority of the injury burden, the majority (36%) lived alone in their own home, 16% lived in their own home with their partner, 10% lived alone but not specified with whom, 4% with younger family, 2% with older relatives, 6% in a residential home and 24% in a nursing home.

**Table 6:** Place of residence in relation to COVID status and neck of femur injury for older patients (65 and above) *Age of 58-80 set for COVID group to allow the inclusion of two patients younger than 65, to allow for a more complete comparison o/a: on admission - patients had confirmed COVID-19

	Non-COVID all trauma		COVID all trauma			Neck of femur fracture (non-COVID)		Neck of femur fracture (COVID)		
Situation of residence	Age 65-80	Age 81+	Age 58-80*	Age 81+	Age 81+ COVID positive o/a	Age 65-80	Age 81+	Age 65-80	Age 81+	Age 81+ COVID positive o/a
Nursing home	0	12	0	7	3	0	9	0	4	2
Residential home	0	2	0	1	1	0	2	0	1	1
Own home - companion not specified	15	4	2	1	2	2	0	0	0	0
Own home - alone	6	21	0	0	1	5	12	0	0	0
Own home - with an elderly parent	1	0	0	0	0	1	0	0	0	0
Own home - with partner	7	5	1	0	0	5	3	0	0	0
Own home - with younger family	1	3	0	0	0	1	1	0	0	0
Home situation unclear	1	1	0	0	0	0	0	0	0	0
TOTAL	31	48	3	9	7	14	27	0	5	3

## Discussion

In this study, we focussed on patients admitted to our institution during the first 30 days of the COVID-19 pandemic lockdown in the UK. This time of global uncertainty and crisis was met by a universal ‘call to arms’ from all involved in healthcare. Similarly, in our unit, we implemented swift changes in how we triaged and managed incoming patients. In summary, the changes we implemented included: i. Suspension of non-emergency and elective work; ii. All staff were redeployed into three ‘squadrons’ with a designated consultant ‘squadron leader’ each day to coordinate all activity in the unit and respond to emerging problems; iii. Work schedules for all in these squadrons were organised so that mixing between teams was minimised to reduce the risk of COVID-19 transmission, with contingency plans in place should any team members fall ill with COVID-19 or have to self-isolate; iv. Daily trauma meetings being held in a larger lecture theatre facilitating 2-metre social distancing, and only those involved with the acute take or operating list attending; v. Maximisation of ‘virtual’ working, including clinical reviews of electronic patient records and telephone consultations for outpatient follow-up where possible, teleconferences instead of meetings, and regional teaching to orthopaedic trainees being delivered via Zoom; vi. Reduction of inpatient stay through increasing early facilitated discharge with the help of orthogeriatric and allied therapy colleagues; cases that required further social work-up stayed temporarily in a local private hospital, which was relatively dormant. Increased trauma day-case operating further reduced stay; vii. Full personal protective equipment (PPE) to be worn for all operations, even where someone has tested negative, to reduce transmission risk; keeping staff healthy and able to work.

At the start of the outbreak, there was a national shortage of testing capability and, as such, COVID PCR testing was rationed; only those deemed at higher risk due to clinical symptoms or proximity to infected cases or radiological or biochemical risk factors were swabbed. Importantly, three of those initially deemed not to have the virus from the ‘low suspicion untested’ group on the above grounds went on to swab positive; having prior knowledge of this, allowing for earlier isolation and treatment would have been useful (Table 1). The patients that were swabbed had a higher mean age and a greater number of classic COVID-19 symptoms. Little was known at the time of asymptomatic COVID-19 disease transmission. Guan et al. found pyrexia on admission in 43%, cough in 67% - with sputum production in 33%, dyspnoea in 18%, and gastrointestinal symptoms in 8.8% [10] When analysing the initial reasons of concern for swab in our positive cohort, 6/12 (50%) had pyrexia, 3/12 (25%) had a cough, 2/12 (16%) were dyspnoeic, and 1/12 (8%) had gastrointestinal (GI) symptoms, which highlights the importance of also considering biochemical and radiological markers (Tables 1-2). Given the nature of asymptomatic transmission and these findings, there is justification for systematic testing of all patients on admission.

Due to the difficulties in isolating a patient in the hospital until their COVID-19 status is known, the data were analysed to see if a pseudo-marker could be identified. Interestingly, lymphopenia had the strongest predictive value of coronavirus (Tables 2,5), seen in 9/12 (75%) of our positive patients. This correlation was also found by Li et al. in 66% of cases, with lower levels directly linked to increased mortality in both these studies [10-11]. As average virus incubation is anywhere between two to seven days (10), we cannot rely on positive symptoms alone to discriminate whether a swab should be taken on admission as could be said of the three ‘low suspicion’ patients at admission going on to test positive (Table 1). Other work also suggests a relationship with raised ferritin levels in up to 66% of COVID positive patients [12], however, this was not routinely performed at our unit, with numbers too small to perform meaningful statistical analysis.

Of note, no overall association was found between gender or discreet comorbidities and risk of contracting coronavirus, however, of those who died of the disease, 2/4 had diabetes with a concurrent diagnosis of cancer, another had cancer alone, and another had complex autoimmune disease managed by immunosuppressant medication (Table 2). This is consistent with a large study from China consisting of 44,672 confirmed cases by Wu et al., which cited both of these conditions as significantly increasing the case fatality ratio present in 7.3% and 5.6% of cases, respectively [13]. There seems to be a trend of increasing risk of contracting the virus according to age: those who contracted the virus were on average 11 years older than patients who were confirmed negative and 33 years older than those who were never tested who had low suspicion of the virus (Table 1), which also concords with other published data [13]. We note no difference in our relatively small cohort in risk of admission for COVID diagnosis in patients admitted from nursing homes, which may have been evident in a larger series (Table 6).

When considering the level of care for those who died of the virus, only one was suitable for escalation to the ITU but died of his symptoms. Two out of four were too frail to be suitable for a level of care beyond supportive measures in the community and died after testing positive for the virus following discharge (IDs 1 and 12). Most of the patients who contracted the virus (8/12), however, recovered and survived beyond 28 days. It is generally reassuring that most patients seem to recover with basic supportive care even if they do have associated comorbidities, offering hope for the typical orthopaedic patient population should a second wave strike. This correlates with data from Wu et al., showing that 80% of patients get a mild form of the disease [13]. The average age of patients who died from COVID-19 (mean 88, SD 1.0) vs. survived (mean 76, SD 12.4) follows other published data, which demonstrate a significantly higher risk of mortality with advancing age (Table 2). Wu et al. saw the death rate increase from the population mean of 2.3% to 14.8% in those aged ≥80, with a fatality rate of 49% in critical cases [13]. When compared with those who died of other causes, we can see that the overall mortality rate was doubled by those who were COVID positive. This may be due to our patient demographic of those with confirmed cases being older and at a higher risk of death than the general population, which would concord with Wu’s data.

On a national UK-wide scale, the first 30 days saw the highest rate of death from coronavirus, as cumulative deaths rose from 359 on March 23 to 21,074 by April 22; the number of daily cases rose from 967 on March 23, to 6199 on April 5 at its peak, and by April 22, had plateaued to 4,451; by May 9, this was 3896 and had begun to fall to 958 per day at the time of writing this report three months after lockdown first commenced [14]. It is, therefore, fair to say that the data we have presented comes from the ‘peak’ of the crisis, and lessons learnt from this would be applicable should there be a ‘second peak’ in the coming months, as it is likely that we could expect a similar number of cases in our unit.

In the early stages of the pandemic, it took up to three days for the swab results to come back, whereas three months later, most results are back within 24 hours, which will be the case if there is a second wave of the virus. Understandably, this had a significant effect on time to theatre, which was increased by an average of one day and affected 4/8 positive cases who needed an operation (Table 2). We also noticed that there was a significant increase in the average case time due to all of the new procedures adopted to mediate the risk to operating theatre staff; although having gone through this learning process, efficiency has improved overall. Despite this, however, any cases in patients with ‘unknown’ status (such as emergencies) and COVID-positive patients still require extra precautions in a ‘hot’ theatre shared with other surgical specialities, which slows everything down. Patients admitted overnight often have to wait at least an extra day by the time their result has come back, for their operation in an orthopaedic ‘cold’ theatre, especially as levels of trauma continue to approach normal ‘pre-COVID’ levels as the current peak of the pandemic subsides. This has broad implications when considering the inherent potential of increased morbidity and complications such as deep vein thrombosis (DVT) and post-operative infection, especially for hip fracture patients, which we can expect to almost double in frequency moving forwards as lockdown restrictions continue to be eased (Table 4), especially given most hip fractures were seen in those living more independently (Table 6), which will require significantly more operations accordingly (Figure 1). Since the first wave, we have also changed the PCR assay tests we use to detect COVID-19, which has improved the efficiency and reliability of tests. These issues need to be taken into account when planning ahead for operating capacity and patient flow in the future, especially if there is a ‘second peak’ of infection and operating theatres are once again converted into temporary intensive care units as they were during the first six weeks of the pandemic.

The effects of the lockdown are apparent from the nature of the injuries, for example, there was less than half the number of polytrauma compared to the previous year (Table 4). Whereas other reasons for admissions, which are less activity-related, such as septic arthritis, remained consistent. The proportion of total hip replacements carried out for neck of femur fractures during the first 30 days of COVID was 4% as compared to 16% over the same time frame the previous year (Figure 1). The study findings are similar to another UK level 1 trauma centre [15] and others internationally [16-17]. The British Orthopaedic Association guidance sought to reduce total hip replacement for trauma and do less internal fixation where possible [18]. Accordingly, we followed this advice that contributed to a 78% reduction in open reduction and internal fixation (ORIF) procedures of all joints (Figure 1). We have significantly increased the number of patients we manage in self-removable soft-casts, splints and walker boots, which can be removed at home with either telephone follow-up by ourselves, physiotherapy or no follow-up at all, and note that similar practices have been adopted elsewhere [19].

Overall, based on our experience, the following can be deducted and considered should there be a second COVID wave:

1. Reduction of trauma volume

This allows for a significantly increased consultant and registrar manpower for acute services and cross-cover on wards, and a higher concentration of senior decision-makers “at the front door” thus reducing any unnecessary admissions or follow-up arrangements.

2. Appropriate allocation of manpower to run a safe, efficient service

With elective orthopaedic activity suspended in order to expand capacity for acute patients, an orthopaedic consultant-led team can also be formed to run the minor injuries unit (MIU), taking this type of work from the emergency department (ED) in order to allow them to focus on sick medical patients rather than trauma. Senior input allows the early assessment of suitability for resuscitation and appropriate escalation of level of care in the ED setting before the patient is admitted, with early senior input using the Rockwood frailty score to guide decision-making, which is inbuilt into our electronic health record system, facilitating the appropriate rationing of intensive care resources for those who will benefit most.

3. Expedited COVID swab test processing allows early, appropriate de-escalation of isolation and improved time to theatre.

4. Increased testing capacity to all admitted patients identifies cases that would otherwise have been missed.

5. Expect approximately 12% of admitted patients to be COVID positive (12/98), and up to 33% of these patients to die - significantly increasing mortality rate, allowing strategic planning and appropriate resource allocation.

6. The vast majority of COVID-positive patients will not require ITU care and can be appropriately managed at the ward level.

7. Consider the use of lymphopenia as a pseudo marker for COVID-19 in separating patients on admission before COVID-19 swab results are available.

8. Remote working, both for clinical meetings and patient interactions, was more successful than anticipated, improving efficiency, and much of this could be brought into effect going forwards, following appropriate review and consultation with all involved.

Limitations of the study

We have presented data typical for orthopaedic admissions to a major trauma centre, however, overall, there are a relatively small number of patients as far as COVID-19 +ve is concerned, as well as COVID-19 deaths. Also, following the initial phase of the pandemic, our armamentarium has already improved - following the lessons learnt, we are now better equipped with PPE, and swab testing volume and turnaround have continued to improve. We, therefore, expect and hope that if we were to perform the same analysis following the second wave, the results would be better.

## Conclusions

The effect of the strict lockdown measures of the government induced a reduced volume of patients and affected the nature of orthopaedic injuries. In addition, it changed our practice towards doing fewer operative interventions to minimise unnecessary patient contact, which could increase the risk of COVID transmission. Lymphopenia is a potentially good surrogate marker of COVID-19, which could be used in patient separation on admission to hospital. Above all, we trust that the lessons learnt during this period have given us a chance to reflect on ways to further improve what we do and be well-steeled to face a ‘second wave’ if and when it should come our way.
